# ﻿Eight new spider species of *Belisana* Thorell, 1898 (Araneae, Pholcidae), with an updated overview of *Belisana* species from Yunnan, China

**DOI:** 10.3897/zookeys.1202.121633

**Published:** 2024-05-27

**Authors:** Ludan Zhang, Zheng Wu, Shuqiang Li, Zhiyuan Yao

**Affiliations:** 1 College of Life Science, Shenyang Normal University, Shenyang 110034, Liaoning, China Shenyang Normal University Shenyang China; 2 Institute of Zoology, Chinese Academy of Sciences, Beijing 100101, China Institute of Zoology, Chinese Academy of Sciences Beijing China

**Keywords:** Biodiversity, checklist, daddy-long-legs, fogging, invertebrate, morphology, taxonomy

## Abstract

In this study, eight new species are described from the subtropical parts of Yunnan Province in southwestern China: *Belisanahonghe* Zhang, Li & Yao, **sp. nov.** (♂♀), *B.jiuxiang* Zhang, Li & Yao, **sp. nov.** (♂♀), *B.lincang* Zhang, Li & Yao, **sp. nov.** (♂♀), *B.luxi* Zhang, Li & Yao, **sp. nov.** (♂♀), *B.tengchong* Zhang, Li & Yao, **sp. nov.** (♂♀), *B.tongi* Zhang, Li & Yao, **sp. nov.** (♂♀), *B.yongsheng* Zhang, Li & Yao, **sp. nov.** (♂), and *B.yunnan* Zhang, Li & Yao, **sp. nov.** (♂♀). They add up to a total of 31 *Belisana* species from Yunnan in an updated list provided in this paper.

## ﻿Introduction

The family Pholcidae C.L. Koch, 1850 is a highly diverse spider group with 1,969 extant species in 97 genera (WSC 2024). *Belisana* Thorell, 1898 had been a monotypic genus for more than 100 years. Huber (2005) was the first arachnologist to revise this genus: he transferred nine species from *Spermophora* Hentz, 1841 to *Belisana* and described 53 new species from Southeast Asia and northern Australia. Yao and Li (2013) and Yao et al. (2013, 2015) redescribed the type species *B.tauricornis* Thorell, 1898 based on type material from Myanmar, and reported 19 new species from Laos and Vietnam. In China, several researchers have also reported a large number of new species of *Belisana*. For instance, Zhang and Peng (2011) described 11 new species from southern China (in the provinces of Yunnan, Guizhou, Guangxi, Guangdong, and Hainan). Yao and his colleagues identified 17 species from Xishuangbanna in Yunnan, including 15 new species (e.g., Yao et al. 2018; Zhao et al. 2023); they also described 11 new species from Tibet, Sichuan, Guizhou, Guangxi, Guangdong, and Fujian (Zhu et al. 2020a, b; Yang et al. 2023). To date, 62 species have been recorded in southern China (Yang et al. 2023; Zhao et al. 2023; WSC 2024). Altogether, there are currently 148 species of *Belisana* globally, making it the second-largest genus in Pholcidae (WSC 2024). They are distributed mainly in southern China, and in the Indo-Malayan and Australasian regions (Huber 2005; Zhu et al. 2020a; WSC 2024). They occupy a variety of micro-habitats, e.g., under rocks, in caves, on the underside of leaves, among leaf litter, and amidst foliage in the canopy (Huber 2005; Yao and Li 2013; Yao et al. 2015).

**Figure 1.**
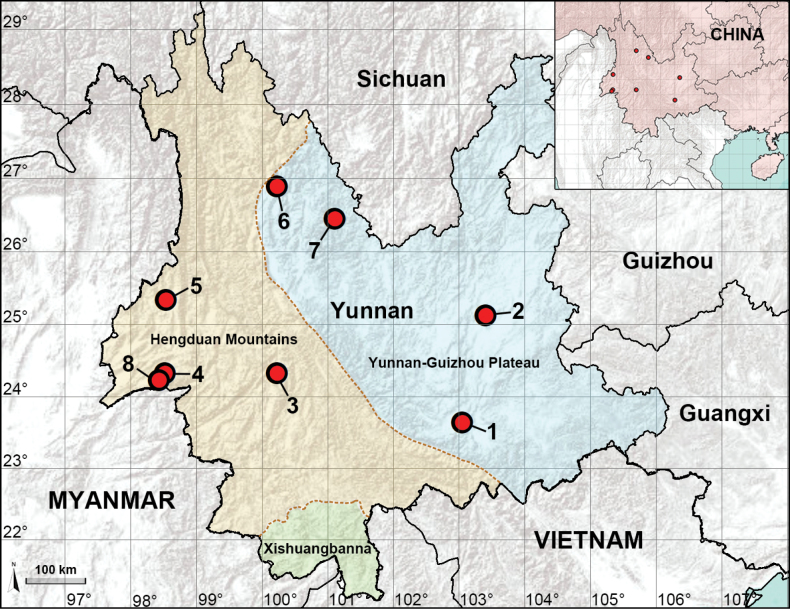
Distribution records of the new *Belisana* species from Yunnan, China **1***Belisanahonghe* sp. nov. **2***B.jiuxiang* sp. nov. **3***B.lincang* sp. nov. **4***B.luxi* sp. nov. **5***B.tengchong* sp. nov. **6***B.tongi* sp. nov. **7***B.yongsheng* sp. nov. **8***B.yunnan* sp. nov.

Currently, 23 species of *Belisana* have been recorded from Yunnan in southwestern China (WSC 2024). Most of them (19 species) were described from tropical Xishuangbanna in southern Yunnan (Huber 2005; Zhang and Peng 2011; Zhao et al. 2023). Another four species were recorded in the subtropical parts of Yunnan: two in the Hengduan Mountains (western Yunnan) and another two on the Yunnan-Guizhou Plateau (eastern Yunnan) (Huber 2005; Zhang and Peng 2011). This work aims to describe the newly discovered species from these two subtropical sites (Fig. 1) and to provide an updated overview of the diversity of *Belisana* species in Yunnan.

## ﻿Materials and methods

Specimens were examined and measured with a Leica M205 C stereomicroscope. Left male palps were photographed. Epigynes were photographed before dissection. Vulvae were photographed after being treated in a 10% warm potassium hydroxide (KOH) solution to dissolve soft tissues. Images were captured with a Canon EOS 750D wide zoom digital camera (24.2 megapixels) mounted on the stereomicroscope mentioned above and assembled using Helicon Focus v. 3.10.3 image stacking software (Khmelik et al. 2005). Drawings were done with Procreate 5.0.2 (Savage Interactive Pty. Ltd.). All measurements are given in millimeters (mm). Leg measurements are shown as: total length (femur, patella, tibia, metatarsus, and tarsus). Leg segments were measured on their dorsal sides. The distribution map was generated with ArcGIS v. 10.2 (ESRI Inc.). The specimens studied are preserved in 75% ethanol and deposited in the Institute of Zoology, Chinese Academy of Sciences (**IZCAS**) in Beijing, China.

Terminology and taxonomic descriptions follow Huber (2005) and Yao et al. (2015). The following abbreviations are used in the descriptions: **ALE** = anterior lateral eye, **AME** = anterior median eye, **PME** = posterior median eye, L/d = length/diameter; used in the illustrations: **aa** = anterior arch, **b** = bulb, **ba** = bulbal apophysis, **da** = distal apophysis, **e** = embolus, ep = epigynal pocket, **f** = flap, pa = proximo-lateral apophysis, **pp** = pore plate, pr = procursus.

## ﻿Taxonomy

### ﻿Family Pholcidae C.L. Koch, 1850

**Subfamily Pholcinae C.L. Koch, 1850**

#### ﻿Genus Belisana

Taxon classificationAnimaliaAraneaePholcidae

Thorell, 1898

69B3289C-E687-5284-8014-27A6F1B1D5BC

##### Type species.

*Belisanatauricornis* Thorell, 1898.

##### Notes.

A total of eight new species from Yunnan were recognized. Of these, *B.honghe* sp. nov., *B.jiuxiang* sp. nov., *B.tongi* sp. nov., and *B.yongsheng* sp. nov. are from the Yunnan-Guizhou Plateau; *B.lincang* sp. nov., *B.luxi* sp. nov., *B.tengchong* sp. nov., and *B.yunnan* sp. nov. are from the Hengduan Mountains (Fig. 1). A list of all *Belisana* species from Yunnan is provided in Table 1.

**Table 1.** A list of the *Belisana* species from Yunnan, China.

**The Hengduan Mountains**	3. *B.chenjini* Yao & Li, 2018
1. *B.lincang* sp. nov.	4. *B.chuandiani* Li, Zheng & Yao, 2023
2. *B.luxi* sp. nov.	5. *B.daxiangi* Li, Zheng & Yao, 2023
3. *B.nujiang* Huber, 2005	6. *B.dian* Yao & Li, 2018
4. *B.pianma* Huber, 2005	7. *B.fengzheni* Li, Zheng & Yao, 2023
5. *B.tengchong* sp. nov.	8. *B.gupian* Yao & Li, 2018
6. *B.yunnan* sp. nov.	9. *B.lata* Zhang & Peng, 2011
**The Yunnan-Guizhou Plateau**	10. *B.menghai* Yao & Li, 2019
1. *B.erromena* Zhang & Peng, 2011	11. *B.mengla* Yao & Li, 2020
2. *B.honghe* sp. nov.	12. *B.menglun* Yao & Li, 2020
3. *B.jiuxiang* sp. nov.	13. *B.mengyang* Yao & Li, 2020
4. *B.tongi* sp. nov.	14. *B.rollofoliolata* (Wang, 1983)
5. *B.yangi* Zhang & Peng, 2011	15. *B.schwendingeri* Huber, 2005
6. *B.yongsheng* sp. nov.	16. *B.xiaolongha* Zhu & Li, 2021
**Xishuangbanna**	17. *B.xishuangbanna* Yao & Li, 2019
1. *B.bubeng* Zhu & Li, 2021	18. *B.yangxiaodongi* Yao & Li, 2018
2. *B.cas* Yao & Li, 2018	19. *B.zhengi* Yao, Pham & Li, 2015

#### ﻿ Belisana honghe

Taxon classificationAnimaliaAraneaePholcidae

Zhang, Li & Yao sp. nov.

6490B690-9CBC-55C3-A67E-998A47145E15

https://zoobank.org/4945FF76-8093-4849-AEBC-3F7715FA09B0

**Figure 2.**
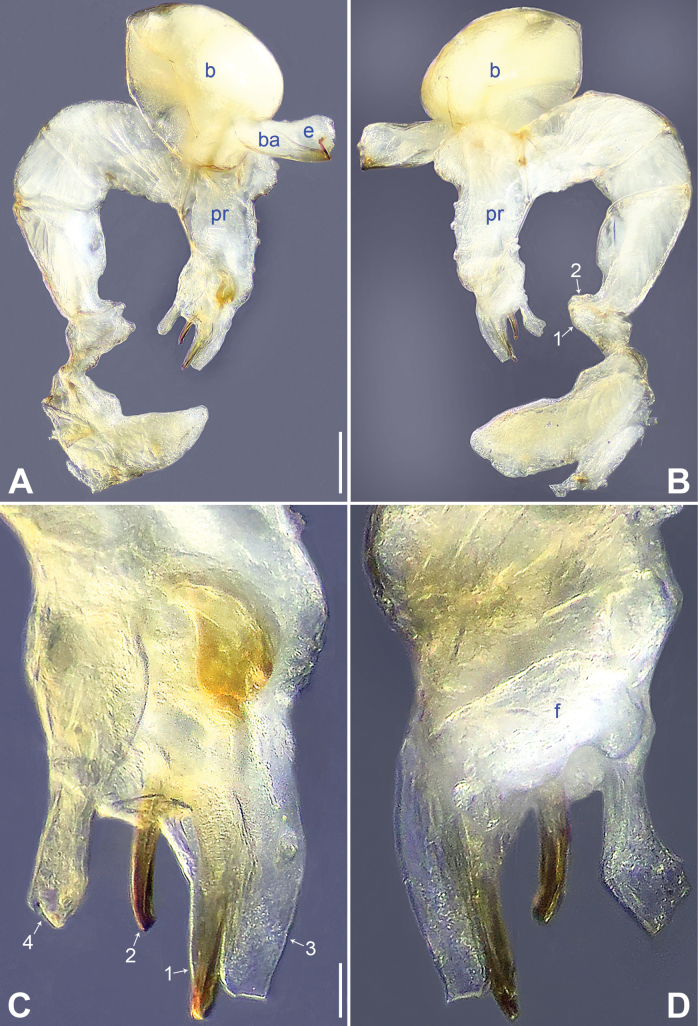
*Belisanahonghe* sp. nov., holotype male **A, B** palp (**A** prolateral view **B** retrolateral view, arrow 1 points at retrolateral apophysis, arrow 2 points at ventral apophysis) **C, D** distal part of procursus (**C** prolateral view, arrow 1 points at sclerotized prolatero-distal apophysis, arrow 2 points at sclerotized distal apophysis, arrow 3 points at dorso-distal membranous process, arrow 4 points at ventro-subdistal membranous process **D** retrolateral view). Abbreviations: b = bulb, ba = bulbal apophysis, e = embolus, f = flap, pr = procursus. Scale bars: 0.10 (**A, B**); 0.02 (**C, D**).

**Figure 3.**
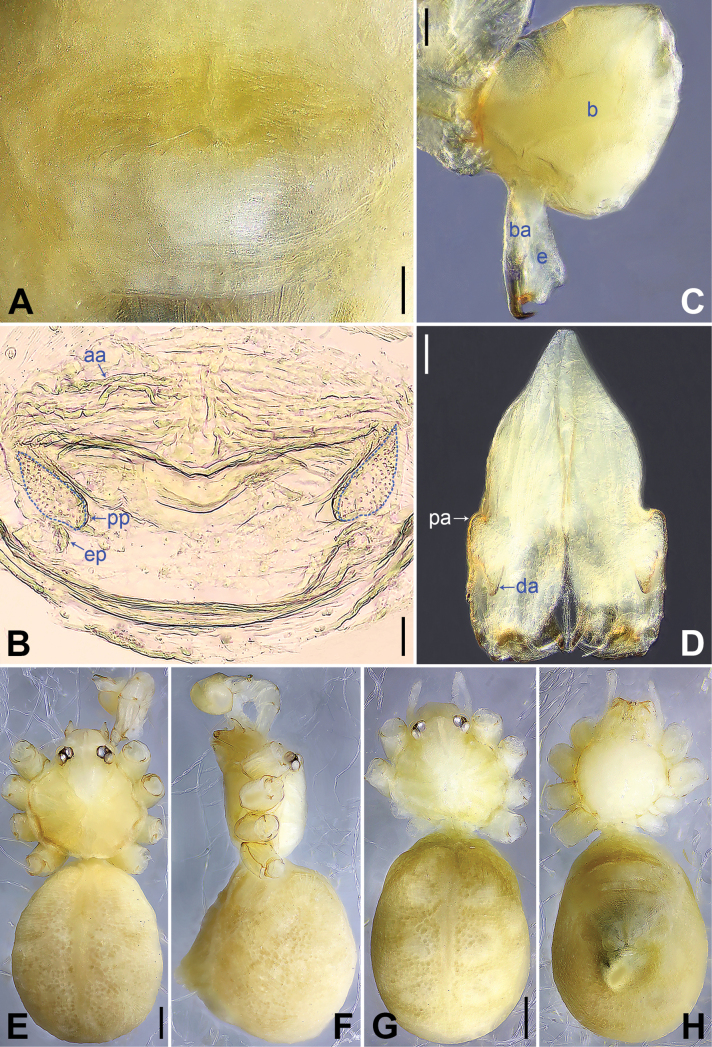
*Belisanahonghe* sp. nov., holotype male (**C–F**) and paratype female (**A, B, G, H**) **A** epigyne, ventral view **B** vulva, dorsal view **C** bulb, prolateral view **D** chelicerae, frontal view **E–H** habitus (**E, G** dorsal view **F** lateral view **H** ventral view). Abbreviations: aa = anterior arch, b = bulb, ba = bulbal apophysis, da = distal apophysis, e = embolus, ep = epigynal pocket, pa = proximo-lateral apophysis, pp = pore plate. Scale bars: 0.05 (**A–D**); 0.20 (**E–H**).

Figs 2 , 3 , 18A, B , 20A, B

##### Type material.

***Holotype*** ♂ (IZCAS-Ar44949) and ***paratypes*** 2♂ (IZCAS-Ar44950–51) 3♀ (IZCAS-Ar44952–54), Yanzi Cave (23°38.220'N, 103°3.200'E, 1080 m), Mawangzhuang, Miandian Town, Jianshui County, Honghe, **Yunnan**, **China**, 29/03/2007, J Liu & Y Lin leg.

##### Etymology.

The specific name refers to the type locality, which is a noun in apposition.

##### Diagnosis.

The new species resembles *B.cheni* Yao, Pham & Li, 2015 (Yao et al. 2015: 5, figs 4A–D, 5A–G, 6A–E) by having similar male chelicerae, bulbal apophyses, and epigyne (Figs 3A, C, D, 20A), but can be distinguished by differences in males: procursus with nearly trapezoidal dorso-distal membranous process (arrow 3 in Figs 2C, 18A vs half-round) and long (as long as dorso-distal membranous process), distally widened ventro-subdistal membranous process (arrow 4 in Figs 2C, 18A vs short and distally pointed); differences in females: pore plates anteriorly pointed and posteriorly wide and blunt (Figs 3B, 20B vs narrow).

##### Description.

**Male** (***holotype***): Total length 1.66 (1.74 with clypeus), carapace 0.63 long, 0.67 wide, opisthosoma 1.03 long, 0.84 wide. Leg I: 19.02 (5.05, 0.26, 5.00, 7.05, 1.66), leg II: 14.38 (3.96, 0.25, 3.80, 5.15, 1.22), leg III missing, leg IV: 10.65 (3.16, 0.23, 2.53, 3.96, 0.77); tibia I L/d: 89. Eye inter-distances and diameters: PME–PME 0.13, PME 0.07, PME–ALE 0.02, AME absent. Sternum width/length: 0.50/0.46. Habitus as in Fig. 3E, F. Carapace and sternum yellowish, without marks. Legs whitish, without darker rings. Opisthosoma yellowish, without spots. Thoracic furrow absent. Clypeus unmodified. Chelicerae (Fig. 3D) with a pair of proximo-lateral apophyses and a pair of distal apophyses (distance between tips: 0.19). Palp as in Fig. 2A, B; trochanter with ventral apophysis (arrow 2 in Fig. 2B) and prolateral apophysis (arrow 1 in Fig. 2B); procursus simple proximally but complex distally, with sclerotized prolatero-distal apophysis (arrow 1 in Figs 2C, 18A), sclerotized distal apophysis (arrow 2 in Figs 2C, 18A), dorso-distal membranous process (arrow 3 in Figs 2C, 18A), ventro-subdistal membranous process (arrow 4 in Figs 2C, 18A), and retrolateral flap (Figs 2D, 18B); bulb (Fig. 3C) with hooked apophysis and simple embolus. Retrolateral trichobothria on tibia I at 9% proximally; legs with short vertical setae on metatarsi; tarsus I with 20 distinct pseudosegments.

**Female** (***paratype***, IZCAS-Ar44952): Similar to male, habitus as in Fig. 3G, H. Total length 1.50 (1.62 with clypeus), carapace 0.49 long, 0.55 wide, opisthosoma 1.01 long, 0.80 wide; tibia I: 3.60; tibia I L/d: 61. Eye inter-distances and diameters: PME–PME 0.14, PME 0.06, PME–ALE 0.02, AME absent. Sternum width/length: 0.47/0.44. Epigyne (Figs 3A, 20A) simple and flat, with a pair of postero-lateral pockets 0.32 apart (arrow ep in Figs 3B, 20B, invisible in Figs 3A, 20A). Vulva (Figs 3B, 20B) with ridge-shaped anterior arch and a pair of anteriorly pointed and posteriorly wide and blunt pore plates.

##### Variation.

Tibia I in one male paratype (IZCAS-Ar44950) (leg I missing in IZCAS-Ar44951): 5.64. Tibia I in the other two female paratypes (IZCAS-Ar44953–54): 4.10, 4.25.

##### Habitat.

The species was found in the dark zone inside the cave.

##### Distribution.

China (Yunnan, type locality; Fig. 1).

#### ﻿ Belisana jiuxiang

Taxon classificationAnimaliaAraneaePholcidae

Zhang, Li & Yao sp. nov.

B6A9BEA5-3AF2-5F6D-A8B4-C5D3C57E57CD

https://zoobank.org/734AE954-74BA-4B1B-81CE-1698150AEF86

**Figure 4.**
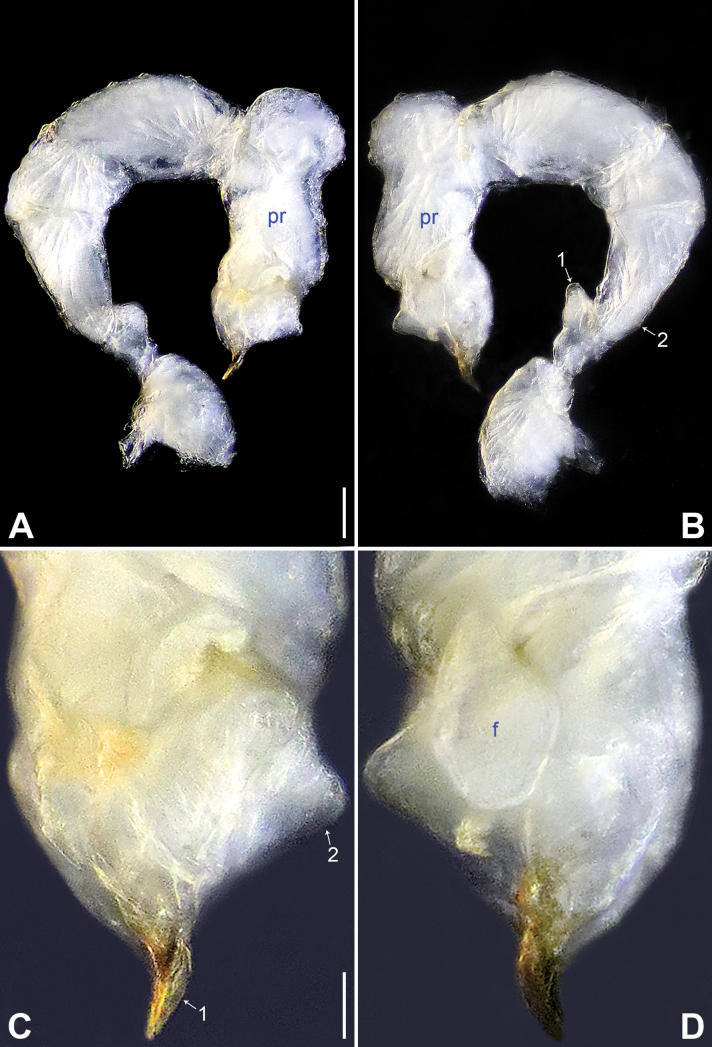
*Belisanajiuxiang* sp. nov., holotype male **A, B** palp (**A** prolateral view **B** retrolateral view, arrow 1 points at ventral apophysis, arrow 2 points at retrolatero-proximal protrusion) **C, D** distal part of procursus (**C** prolateral view, arrow 1 points at sclerotized distal apophysis, arrow 2 points at dorso-subdistal membranous process **D** retrolateral view). Abbreviations: f = flap, pr = procursus. Scale bars: 0.10 (**A, B**); 0.02 (**C, D**).

**Figure 5.**
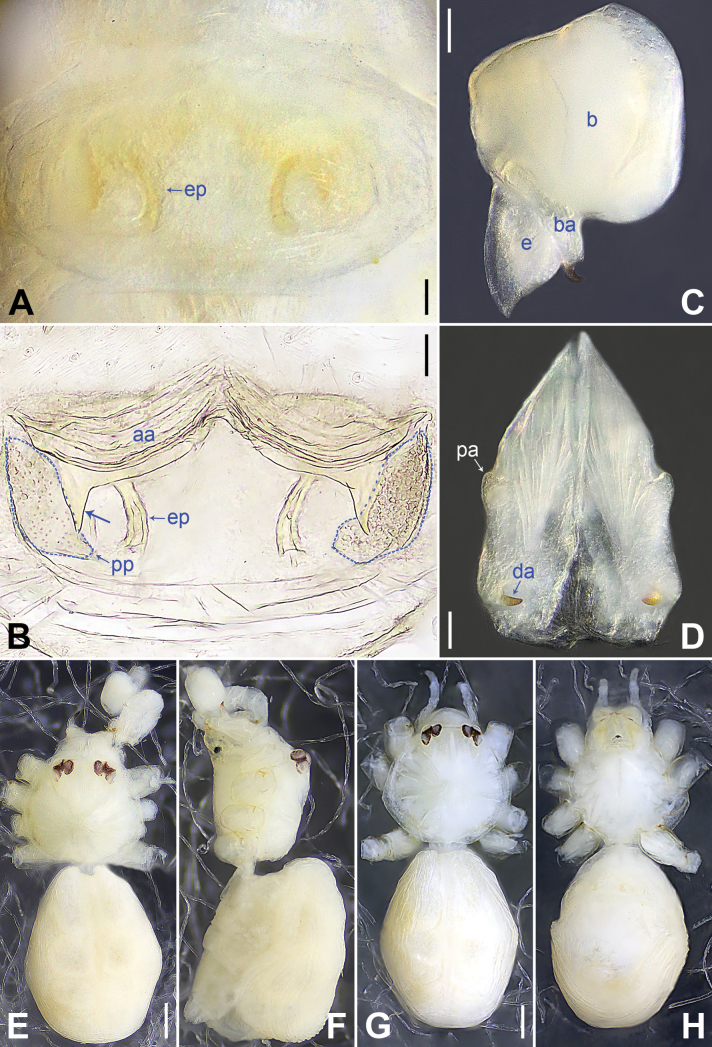
*Belisanajiuxiang* sp. nov., holotype male (**C–F**) and paratype female (**A, B, G, H**) **A** epigyne, ventral view **B** vulva, dorsal view, arrow points at lateral sclerite **C** bulb, prolateral view **D** chelicerae, frontal view **E–H** habitus (**E, G** dorsal view **F** lateral view **H** ventral view). Abbreviations: aa = anterior arch, b = bulb, ba = bulbal apophysis, da = distal apophysis, e = embolus, ep = epigynal pocket, pa = proximo-lateral apophysis, pp = pore plate. Scale bars: 0.05 (**A–D**); 0.20 (**E–H**).

Figs 4 , 5 , 18C, D , 20C, D

##### Type material.

***Holotype*** ♂ (IZCAS-Ar44955) and ***paratypes*** 2♂ (IZCAS-Ar44956–57) 4♀ (IZCAS-Ar44958–61), Sanjiao Cave (25°8.059'N, 103°23.969'E, 1795 m), Jiuxiang Town, Yiliang County, Kunming, **Yunnan**, **China**, 08/04/2007, J Liu & Y Lin leg.

##### Etymology.

The specific name refers to the type locality, which is a noun in apposition.

##### Diagnosis.

The new species resembles *B.tianlinensis* Zhang & Peng, 2011 (Zhang and Peng 2011: 65, fig. 10A–G) by having similar male chelicerae and bulbal apophyses (Fig. 5C, D), but can be distinguished by differences in males: clypeus unmodified (Fig. 5E vs with front apophysis), procursus with pointed, sclerotized distal apophysis (arrow 1 in Figs 4C, 18C vs with dorso-distal spine) and dorso-subdistal membranous process (arrow 2 in Figs 4C, 18C vs absent); differences in females: epigyne with median pockets (Figs 5A, B, 20C, D vs lateral), pore plates quadrilateral (Figs 5B, 20D vs nearly triangular).

##### Description.

**Male** (***holotype***): Total length 1.64 (1.74 with clypeus), carapace 0.63 long, 0.67 wide, opisthosoma 1.01 long, 0.80 wide. Leg I: 14.84 (3.86, 0.33, 3.80, 5.15, 1.70), leg II: 9.62 (2.64, 0.26, 2.45, 3.17, 1.10), leg III: 6.82 (2.03, 0.19, 1.66, 2.28, 0.66), leg IV: 8.79 (2.56, 0.26, 2.25, 2.94, 0.78); tibia I L/d: 56. Eye inter-distances and diameters: PME–PME 0.13, PME 0.07, PME–ALE 0.02, AME absent. Sternum width/length: 0.53/0.48. Habitus as in Fig. 5E, F. Carapace and sternum yellowish, without marks. Legs whitish, without darker rings. Opisthosoma yellowish, without spots. Thoracic furrow absent. Clypeus unmodified. Chelicerae (Fig. 5D) with a pair of proximo-lateral apophyses and a pair of curved distal apophyses (distance between tips: 0.16). Palp as in Fig. 4A, B; trochanter with ventral apophysis (as long as wide, arrow 1 in Fig. 4B); femur with tiny retrolatero-proximal protrusion (arrow 2 in Fig. 4B); procursus simple proximally but complex distally, with pointed, sclerotized distal apophysis (arrow 1 in Figs 4C, 18C), dorso-subdistal membranous process (arrow 2 in Figs 4C, 18C), and nearly elliptic retrolateral flap (Figs 4D, 18D); bulb (Fig. 5C) with hooked apophysis and simple embolus. Retrolateral trichobothria on tibia I at 8% proximally; legs with short vertical setae on metatarsi; tarsus I with 19 distinct pseudosegments.

**Female** (***paratype***, IZCAS-Ar44958): Similar to male, habitus as in Fig. 5G, H. Total length 1.82 (1.92 with clypeus), carapace 0.73 long, 0.80 wide, opisthosoma 1.09 long, 0.83 wide; tibia I: 2.37; tibia I L/d: 36. Eye inter-distances and diameters: PME–PME 0.14, PME 0.06, PME–ALE 0.02, AME absent. Sternum width/length: 0.61/0.60. Epigyne (Figs 5A, 20C) simple and flat, anteriorly slightly sclerotized, with a pair of median pockets 0.15 apart. Vulva (Figs 5B, 20D) with ridge-shaped anterior arch bearing a pair of angular lateral sclerites (arrow in Figs 5B, 20D) and a pair of quadrilateral pore plates.

##### Variation.

Tibia I in two male paratypes (IZCAS-Ar44956–57): 3.91, 4.36. Tibia I in the other three female paratypes (IZCAS-Ar44959–61): 2.84, 3.72, 3.78.

##### Habitat.

The species was found in the dark zone inside the cave.

##### Distribution.

China (Yunnan, type locality; Fig. 1).

#### ﻿ Belisana lincang

Taxon classificationAnimaliaAraneaePholcidae

Zhang, Li & Yao sp. nov.

D72D17B6-CC22-5D3C-BB6C-874820714C8B

https://zoobank.org/7401890D-953B-478C-AFBC-3E8E477689D1

**Figure 6.**
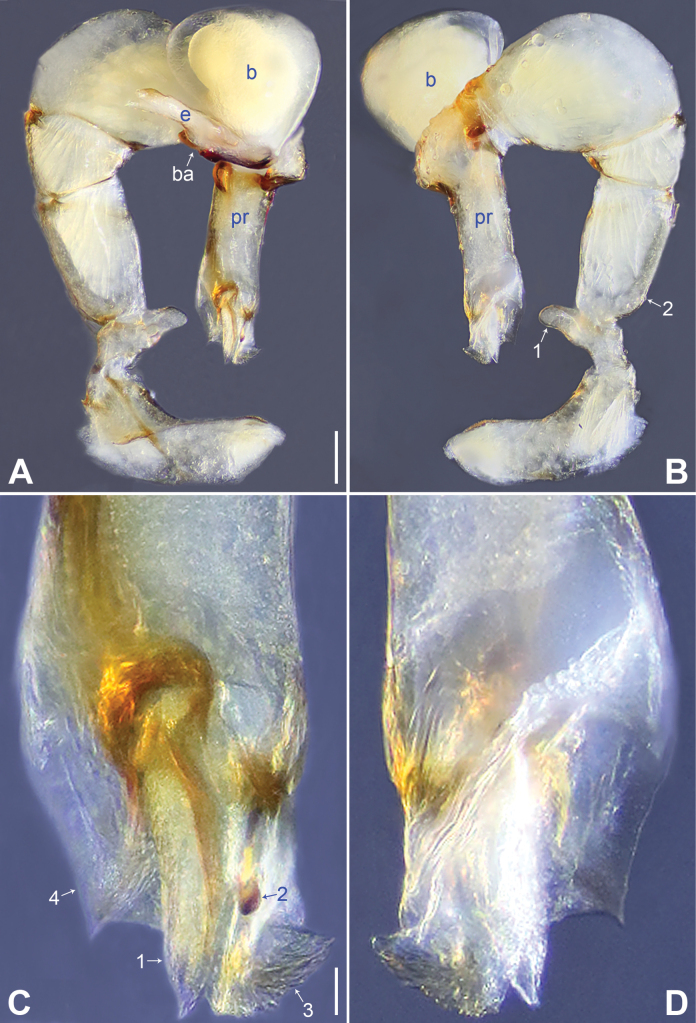
*Belisanalincang* sp. nov., holotype male **A, B** palp (**A** prolateral view **B** retrolateral view, arrow 1 points at ventral apophysis, arrow 2 points at retrolatero-proximal protrusion) **C, D** distal part of procursus (**C** prolateral view, arrow 1 points at prolatero-distal membranous process, arrow 2 points at sclerotized prolatero-distal apophysis, arrow 3 points at dorso-distal membranous lamella, arrow 4 points at ventro-subdistal membranous lamella **D** retrolateral view). Abbreviations: b = bulb, ba = bulbal apophysis, e = embolus, pr = procursus. Scale bars: 0.10 (**A, B**); 0.02 (**C, D**).

**Figure 7.**
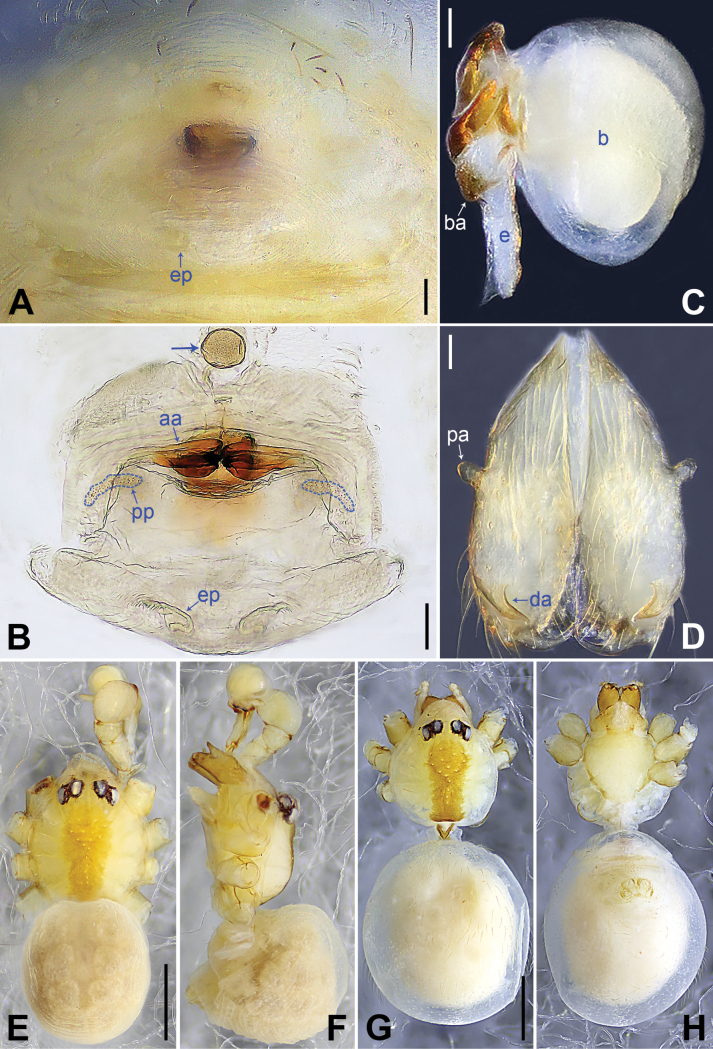
*Belisanalincang* sp. nov., holotype male (**C–F**) and paratype female (**A, B, G, H**) **A** epigyne, ventral view **B** vulva, dorsal view, arrow points at sac-like structure **C** right bulb, prolateral view **D** chelicerae, frontal view **E–H** habitus (**E, G** dorsal view **F** lateral view **H** ventral view). Abbreviations: aa = anterior arch, b = bulb, ba = bulbal apophysis, da = distal apophysis, e = embolus, ep = epigynal pocket, pa = proximo-lateral apophysis, pp = pore plate. Scale bars: 0.05 (**A–D**); 0.50 (**E–H**).

Figs 6 , 7 , 18E, F , 20E, F

##### Type material.

***Holotype*** ♂ (IZCAS-Ar44962) and ***paratype*** 1♀ (IZCAS-Ar44963), Bianfu Cave (24°19.862'N, 100°14.001'E, 1619 m), Yankou Village, Laoxu Town, Yun County, Lincang, **Yunnan**, **China**, 05/08/2010, C Wang, Q Zhao & L Lin leg.

##### Etymology.

The specific name refers to the type locality, which is a noun in apposition.

##### Diagnosis.

The new species resembles *B.phungae* Yao, Pham & Li, 2015 (Yao et al. 2015: 9, figs 19A–D, 20A–G, 21A–E) by having similar male chelicerae, bulbal apophyses, and epigyne (Figs 7A, C, D, 20E), but can be distinguished by differences in males: procursus with dorso-distal membranous lamella (arrow 3 in Figs 6C, 18E vs absent), distally pointed ventro-subdistal membranous lamella (arrow 4 in Figs 6C, 18E vs nearly half-round) and without dorso-subdistal sclerite (Figs 6C, 18E vs present); differences in females: epigynal pockets closer to each other (Figs 7A, B, 20E, F vs widely separated), pore plates curved and narrow (Figs 7B, 20F vs nearly round).

##### Description.

**Male** (***holotype***): Total length 1.78 (1.90 with clypeus), carapace 0.85 long, 0.88 wide, opisthosoma 0.93 long, 0.85 wide. Leg I missing, leg II: 6.69 (1.78, 0.31, 1.62, 2.18, 0.80), leg III: 5.18 (1.42, 0.26, 1.19, 1.68, 0.63), leg IV: 6.70 (1.90, 0.28, 1.70, 2.13, 0.69). Eye inter-distances and diameters: PME–PME 0.08, PME 0.09, PME–ALE 0.03, AME absent. Sternum width/length: 0.61/0.56. Habitus as in Fig. 7E, F. Carapace yellowish, with brown median band; sternum yellowish. Legs whitish, without darker rings. Opisthosoma yellowish, without spots. Thoracic furrow absent. Clypeus unmodified. Chelicerae (Fig. 7D) with a pair of proximo-lateral apophyses and a pair of curved distal apophyses (distance between tips: 0.15). Palp as in Fig. 6A, B; trochanter with ventral apophysis (2× longer than wide, arrow 1 in Fig. 6B); femur with tiny retrolatero-proximal protrusion (arrow 2 in Fig. 6B); procursus simple proximally but complex distally, with prolatero-distal membranous process (arrow 1 in Figs 6C, 18E) bearing narrow lateral sclerite, sclerotized prolatero-distal apophysis (arrow 2 in Figs 6C, 18E), dorso-distal membranous lamella (arrow 3 in Figs 6C, 18E), and ventro-subdistal membranous lamella (arrow 4 in Figs 6C, 18E); bulb (Fig. 7C) with distally angular apophysis and simple embolus.

**Female** (***paratype***, IZCAS-Ar44963): Similar to male, habitus as in Fig. 7G, H. Total length 2.48 (2.62 with clypeus), carapace 0.86 long, 0.86 wide, opisthosoma 1.62 long, 1.31 wide. Leg I: 9.08 (2.33, 0.33, 2.30, 2.97, 1.15); tibia I L/d: 26. Eye inter-distances and diameters: PME–PME 0.09, PME 0.08, PME–ALE 0.02, AME absent. Sternum approximately as wide as long (0.60). Epigyne (Figs 7A, 20E) simple and flat, with dark internal shade and a pair of postero-median pockets 0.05 apart. Vulva (Figs 7B, 20F) with sac-like structure (arrow in Figs 7B, 20F), sclerotized anterior arch, and a pair of curved, long elliptic pore plates (5× longer than wide). Retrolateral trichobothria on tibia I at 9% proximally; legs with short vertical setae on metatarsi; tarsus I with 20 distinct pseudosegments.

##### Habitat.

The species was found in the dark zone inside the cave.

##### Distribution.

China (Yunnan, type locality; Fig. 1).

#### ﻿ Belisana luxi

Taxon classificationAnimaliaAraneaePholcidae

Zhang, Li & Yao sp. nov.

07669D20-542E-5102-A1E8-096AD1457AE7

https://zoobank.org/E3307ED5-995B-42A5-9FC8-84D6F6861A61

**Figure 8.**
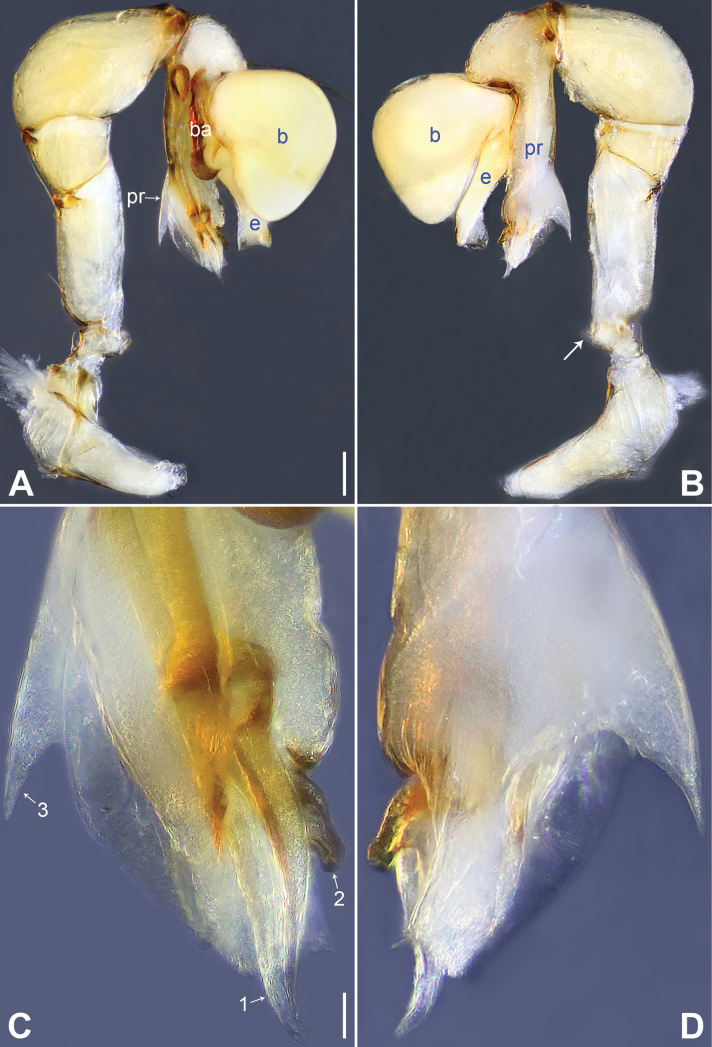
*Belisanaluxi* sp. nov., holotype male **A, B** palp (**A** prolateral view **B** retrolateral view, arrow points at ventral apophysis) **C, D** distal part of procursus (**C** prolateral view, arrow 1 points at prolatero-distal membranous process, arrow 2 points at sclerotized dorso-subdistal apophysis, arrow 3 points at ventro-subdistal membranous process **D** retrolateral view). Abbreviations: b = bulb, ba = bulbal apophysis, e = embolus, pr = procursus. Scale bars: 0.10 (**A, B**); 0.02 (**C, D**).

**Figure 9.**
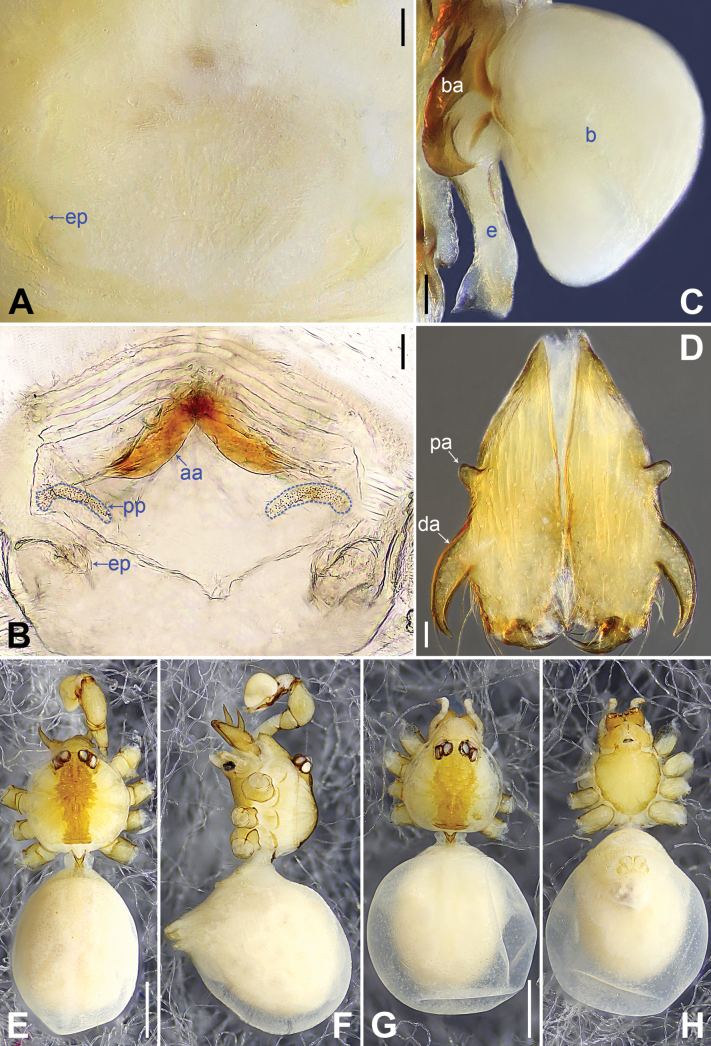
*Belisanaluxi* sp. nov., holotype male (**C–F**) and paratype female (**A, B, G, H**) **A** epigyne, ventral view **B** vulva, dorsal view **C** bulb, prolateral view **D** chelicerae, frontal view **E–H** habitus (**E, G** dorsal view **F** lateral view **H** ventral view). Abbreviations: aa = anterior arch, b = bulb, ba = bulbal apophysis, da = distal apophysis, e = embolus, ep = epigynal pocket, pa = proximo-lateral apophysis, pp = pore plate. Scale bars: 0.05 (**A–D**); 0.50 (**E–H**).

Figs 8 , 9 , 18G, H , 20G, H

##### Type material.

***Holotype*** ♂ (IZCAS-Ar44964) and ***paratypes*** 1♂ (IZCAS-Ar44965) 3♀ (IZCAS-Ar44966–68), Xianfo Cave (24°19.971'N, 98°30.943'E, 1081 m), Mangliu Village, Luxi, Dehong, **Yunnan**, **China**, 03/08/2010, C Wang, Q Zhao & L Lin leg.

##### Etymology.

The specific name refers to the type locality, which is a noun in apposition.

##### Diagnosis.

The new species resembles *B.lincang* sp. nov. (Figs 6, 7, 18E, F, 20E, F) by having similar epigyne and pore plates (Figs 9A, B, 20G, H), but can be distinguished by differences in males: cheliceral distal apophyses pointing downwards (Fig. 9D vs inwards), procursus without dorso-distal membranous lamella (Figs 8C, 18G vs present) and with angular ventro-subdistal membranous process (arrow 3 in Figs 8C, 18G vs distally pointed), bulbal apophysis distally blunt (Fig. 9C vs distally angular); differences in females: epigynal pockets widely separated (Figs 9A, B, 20G, H vs closer to each other), vulva without sac-like structure (Figs 9B, 20H vs present), sclerotized part of anterior arch strongly curved (Figs 9B, 20H vs straight).

##### Description.

**Male** (***holotype***): Total length 2.25 (2.40 with clypeus), carapace 0.83 long, 0.84 wide, opisthosoma 1.42 long, 1.08 wide. Leg I: 15.92 (4.30, 0.33, 4.20, 5.75, 1.34), leg II: 9.31 (2.48, 0.31, 2.35, 3.40, 0.77), leg III: 6.39 (1.64, 0.29, 1.56, 2.25, 0.65), leg IV: 9.07 (2.56, 0.31, 2.35, 3.09, 0.76); tibia I L/d: 53. Eye inter-distances and diameters: PME–PME 0.11, PME 0.10, PME–ALE 0.04, AME absent. Sternum width/length: 0.65/0.63. Habitus as in Fig. 9E, F. Carapace yellowish, with brown median band; sternum yellowish. Legs whitish, without darker rings. Opisthosoma yellowish, without spots. Thoracic furrow absent. Clypeus unmodified. Chelicerae (Fig. 9D) with a pair of proximo-lateral apophyses and a pair of curved distal apophyses (distance between tips: 0.41). Palp as in Fig. 8A, B; trochanter with ventral apophysis (as long as wide, arrow in Fig. 8B); procursus simple proximally but complex distally, with prolatero-distal membranous process (arrow 1 in Figs 8C, 18G) bearing narrow lateral sclerite, sclerotized dorso-subdistal apophysis (arrow 2 in Figs 8C, 18G), and angular ventro-subdistal membranous process (arrow 3 in Figs 8C, 18G); bulb (Fig. 9C) with distally blunt apophysis and simple embolus. Retrolateral trichobothria on tibia I at 14% proximally; legs with short vertical setae on metatarsi; tarsus I with 20 distinct pseudosegments.

**Female** (***paratype***, IZCAS-Ar44966): Similar to male, habitus as in Fig. 9G, H. Total length 2.51 (2.65 with clypeus), carapace 0.89 long, 0.89 wide, opisthosoma 1.62 long, 1.36 wide; tibia I: 2.37; tibia I L/d: 30. Eye inter-distances and diameters: PME–PME 0.09, PME 0.08, PME–ALE 0.03, AME absent. Sternum width/length: 0.61/0.56. Epigyne (Figs 9A, 20G) simple and flat, with a pair of postero-lateral pockets 0.38 apart. Vulva (Figs 9B, 20H) with ridge-shaped, posteriorly sclerotized anterior arch and a pair of curved, long elliptic pore plates (6× longer than wide).

##### Variation.

Tibia I in one male paratype (IZCAS-Ar44965): 3.46. Tibia I in the other two female paratypes (IZCAS-Ar44967–68): 1.78, 2.82.

##### Habitat.

The species was found in the twilight zone (entrance ecotone) of the cave.

##### Distribution.

China (Yunnan, type locality; Fig. 1).

#### ﻿ Belisana tengchong

Taxon classificationAnimaliaAraneaePholcidae

Zhang, Li & Yao sp. nov.

715AB4B4-D68E-591B-BC30-B7284CB2823C

https://zoobank.org/D21705D9-7D42-4F9A-A4E9-27720466DF1D

**Figure 10.**
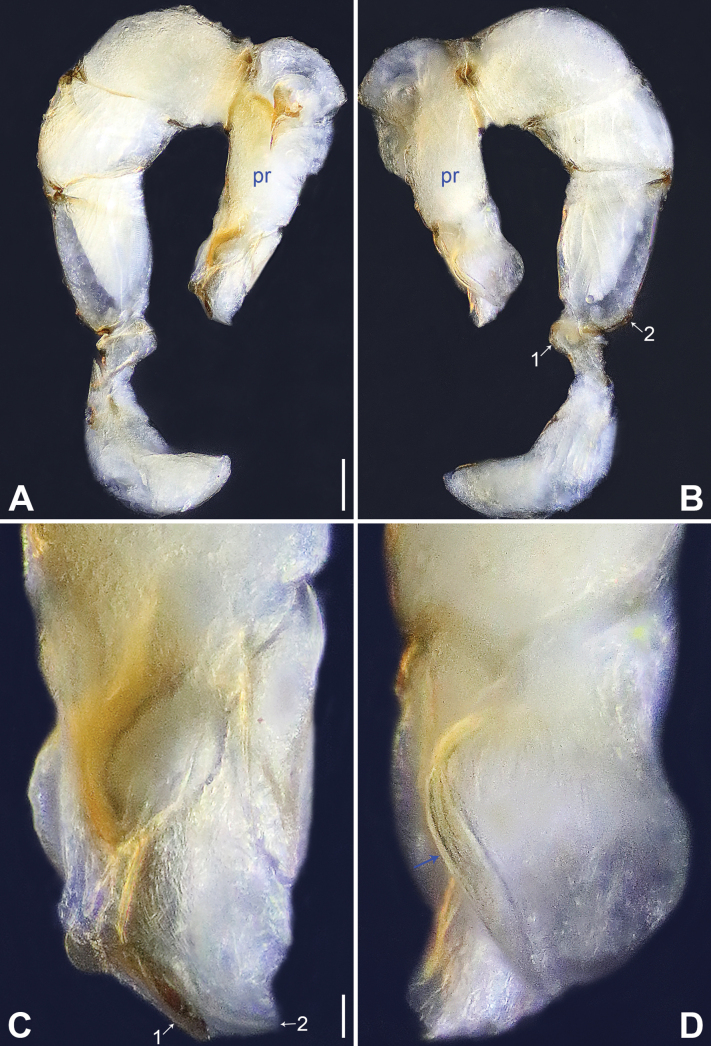
*Belisanatengchong* sp. nov., holotype male **A, B** palp (**A** prolateral view **B** retrolateral view, arrow 1 points at ventral apophysis, arrow 2 points at retrolatero-proximal protrusion) **C, D** distal part of procursus (**C** prolateral view, arrow 1 points at sclerotized prolatero-distal apophysis, arrow 2 points at distal membranous process **D** retrolateral view, arrow points at retrolatero-subdistal membranous process). Abbreviation: pr = procursus. Scale bars: 0.10 (**A, B**); 0.02 (**C, D**).

**Figure 11.**
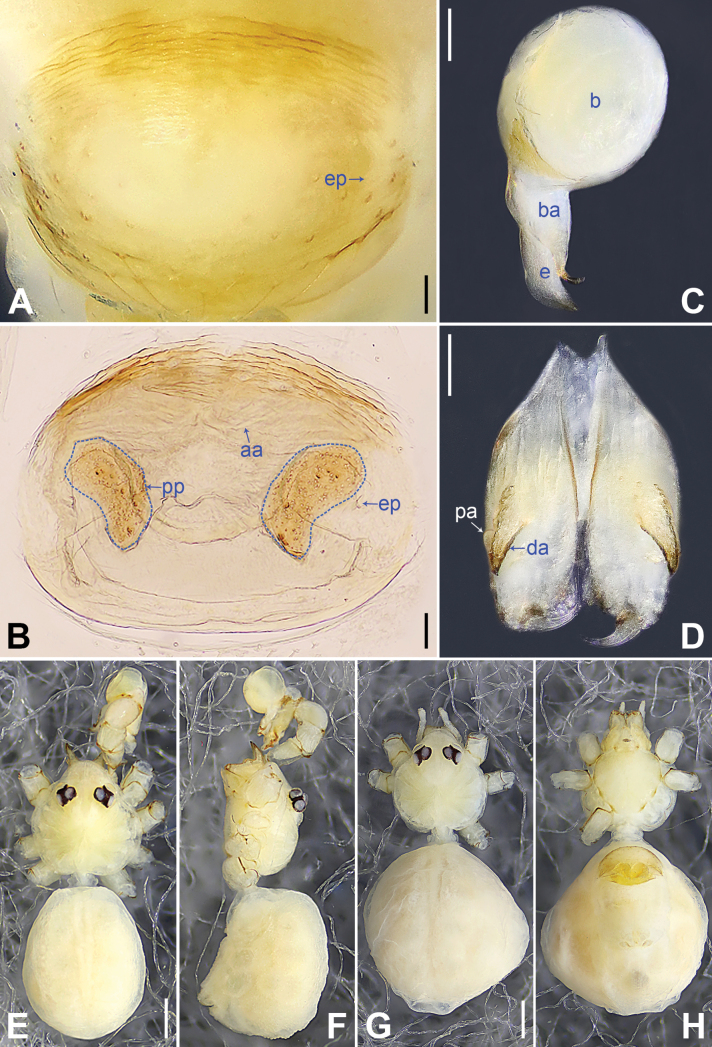
*Belisanatengchong* sp. nov., holotype male (**C–F**) and paratype female (**A, B, G, H**) **A** epigyne, ventral view **B** vulva, dorsal view **C** bulb, prolateral view **D** chelicerae, frontal view **E–H** habitus (**E, G** dorsal view **F** lateral view **H** ventral view). Abbreviations: aa = anterior arch, b = bulb, ba = bulbal apophysis, da = distal apophysis, e = embolus, ep = epigynal pocket, pa = proximo-lateral apophysis, pp = pore plate. Scale bars: 0.05 (**A, B**); 0.10 (**C, D**); 0.30 (**E–H**).

Figs 10 , 11 , 18I, J , 21A, B

##### Type material.

***Holotype*** ♂ (IZCAS-Ar44969) and ***paratypes*** 2♂ (IZCAS-Ar44970–71) 3♀ (IZCAS-Ar44972–74), Kongming Cave (25°20.447'N, 98°32.297'E, 1802 m), Jiangdong Village, Gudong Town, Tengchong County, Baoshan, **Yunnan**, **China**, 18/08/2010, C Wang, Q Zhao & L Lin leg.

##### Etymology.

The specific name refers to the type locality, which is a noun in apposition.

##### Diagnosis.

The new species resembles *B.pianma* Huber, 2005 (Huber 2005: 14, figs 5, 6, 56, 88–92, 112–130) by having similar bulbal apophyses and epigyne (Figs 11A, C, 21A), but can be distinguished by differences in males: clypeus unmodified (Fig. 11E vs with a pair of front apophyses), cheliceral distal apophyses on median part of chelicerae (Fig. 11D vs distal part), procursus without distal spine and dorso-distal sclerite (Figs 10C, 18I vs present), and with angular distal membranous process (arrow 2 in Figs 10C, 18I vs absent); differences in females: pore plates wide and curved (2× longer than wide, Figs 11B, 21B vs narrow, 6× longer than wide).

##### Description.

**Male** (***holotype***): Total length 1.95 (2.08 with clypeus), carapace 0.71 long, 0.85 wide, opisthosoma 1.24 long, 0.93 wide. Leg I: 18.34 (4.75, 0.32, 4.90, 6.67, 1.70), leg II: 12.82 (3.56, 0.26, 3.40, 4.55, 1.05), legs III and IV missing; tibia I L/d: 65. Eye inter-distances and diameters: PME–PME 0.14, PME 0.10, PME–ALE 0.03, AME absent. Sternum width/length: 0.56/0.50. Habitus as in Fig. 11E, F. Carapace and sternum yellowish, without marks. Legs whitish, without darker rings. Opisthosoma yellowish, without spots. Thoracic furrow absent. Clypeus unmodified. Chelicerae (Fig. 11D) with a pair of proximo-lateral apophyses and a pair of curved distal apophyses (distance between tips: 0.28). Palp as in Fig. 10A, B; trochanter with ventral apophysis (as long as wide, arrow 1 in Fig. 10B); femur with tiny retrolatero-proximal protrusion (arrow 2 in Fig. 10B); procursus simple proximally but complex distally, with curved, sclerotized prolatero-distal apophysis (arrow 1 in Figs 10C, 18I), angular distal membranous process (arrow 2 in Figs 10C, 18I), and narrow, curved retrolatero-subdistal membranous process (arrow in Figs 10D, 18J); bulb (Fig. 11C) with hooked apophysis and simple embolus. Retrolateral trichobothria on tibia I on 17% proximally; legs with short vertical setae on metatarsi; tarsus I with 17 distinct pseudosegments.

**Female** (***paratype***, IZCAS-Ar44972): Similar to male, habitus as in Fig. 11G, H. Total length 2.14 (2.26 with clypeus), carapace 0.66 long, 0.76 wide, opisthosoma 1.48 long, 1.34 wide; tibia I: 3.36; tibia I L/d: 47. Eye inter-distances and diameters: PME–PME 0.10, PME 0.10, PME–ALE 0.02, AME absent. Sternum width/length: 0.54/0.50. Epigyne (Figs 11A, 21A) simple and flat, marginally slightly sclerotized, with a pair of lateral pockets 0.30 apart. Vulva (Figs 11B, 21B) with curved anterior arch and a pair of wide, curved pore plates (2× longer than wide) bearing indistinct teeth.

##### Variation.

Tibia I in two male paratypes (IZCAS-Ar44970–71): 5.45, 5.83. Tibia I in the other two female paratypes (IZCAS-Ar44973–74): 3.45, 4.05.

##### Habitat.

The species was found in the dark zone inside the cave.

##### Distribution.

China (Yunnan, type locality; Fig. 1).

#### ﻿ Belisana tongi

Taxon classificationAnimaliaAraneaePholcidae

Zhang, Li & Yao sp. nov.

D224695A-E815-5E5B-9BFE-465CFDB7BD4C

https://zoobank.org/4C53D853-BD6F-4E49-BBC9-DBBE5987F8A5

**Figure 12.**
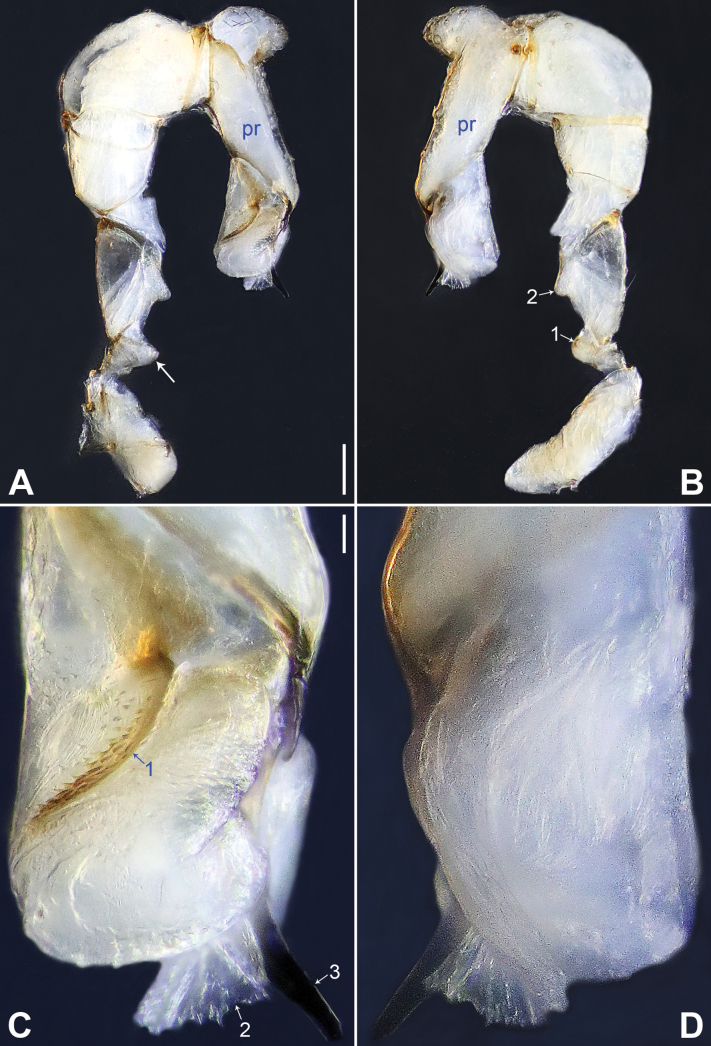
*Belisanatongi* sp. nov., holotype male **A, B** palp (**A** prolateral view, arrow points at ventral apophysis **B** retrolateral view, arrow 1 points at retrolateral apophysis, arrow 2 points at ventral protrusion) **C, D** distal part of procursus (**C** prolateral view, arrow 1 points at prolatero-subdistal part, arrow 2 points at distal membranous process, arrow 3 points at dorso-distal spine **D** retrolateral view). Abbreviation: pr = procursus. Scale bars: 0.10 (**A, B**); 0.02 (**C, D**).

**Figure 13.**
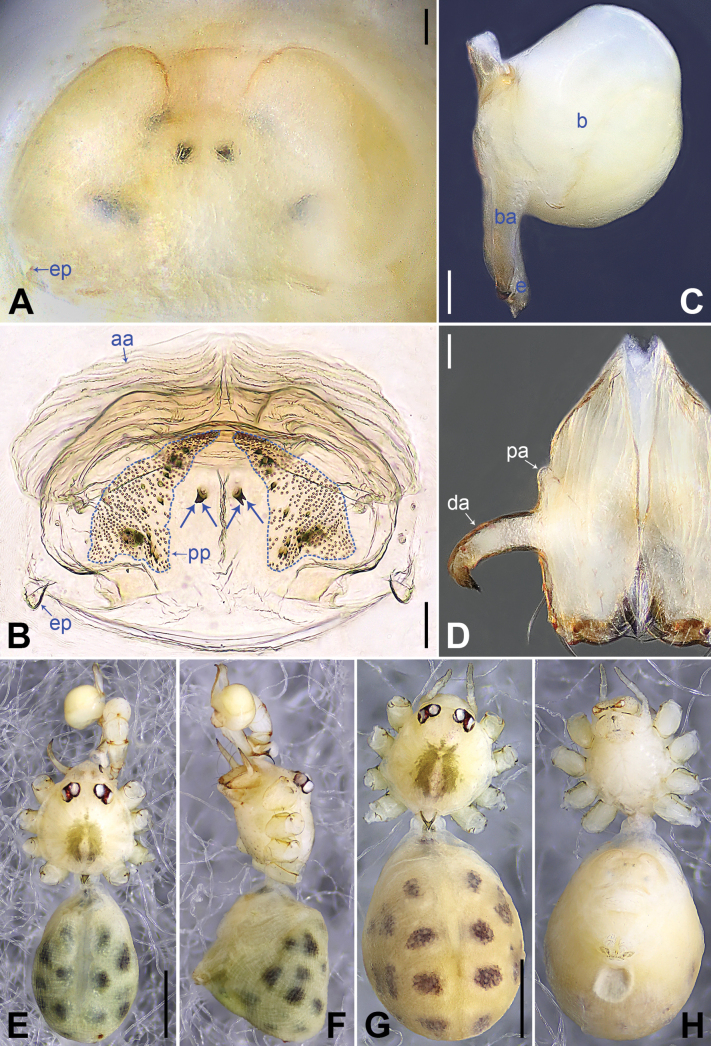
*Belisanatongi* sp. nov., holotype male (**C–F**) and paratype female (**A, B, G, H**) **A** epigyne, ventral view **B** vulva, dorsal view, arrows point at four distinct median teeth **C** bulb, prolateral view **D** chelicerae, frontal view **E–H** habitus (**E, G** dorsal view **F** lateral view **H** ventral view). Abbreviations: aa = anterior arch, b = bulb, ba = bulbal apophysis, da = distal apophysis, e = embolus, ep = epigynal pocket, pa = proximo-lateral apophysis, pp = pore plate. Scale bars: 0.05 (**A–D**); 0.50 (**E–H**).

Figs 12 , 13 , 18K, L , 21C, D

##### Type material.

***Holotype*** ♂ (IZCAS-Ar44975) and ***paratype*s** 4♀ (IZCAS-Ar44976–79), Heilongtan Park (26°53.259'N, 100°14.034'E, 2155 m), Lijiang, **Yunnan**, **China**, 15/07/2021, Y Tong & D Bian leg.

##### Etymology.

The specific name is a patronym in honour of the collector Yanfeng Tong, which is a noun (name) in the genitive case.

##### Diagnosis.

The new species resembles *B.yangi* Zhang & Peng, 2011 (Zhang and Peng 2011: 65, fig. 11A–H) by having similar male chelicerae, bulbal apophyses, and epigyne (Figs 13A, C, D, 21C), but can be distinguished by differences in males: procursus with distal membranous process (arrow 2 in Figs 12C, 18K vs absent) and dorso-distal spine (arrow 3 in Figs 12C, 18K vs absent); differences in females: epigyne with posterior pockets (Figs 13A, B, 21C, D vs anterior), vulva with four distinct median teeth (arrows in Figs 13B, 21D vs absent), pore plates nearly triangular (Figs 13B, 21D vs elliptic).

##### Description.

**Male** (***holotype***): Total length 1.96 (2.08 with clypeus), carapace 0.71 long, 0.75 wide, opisthosoma 1.25 long, 0.80 wide. Leg I: 12.57 (3.30, 0.32, 3.22, 4.50, 1.23), leg II: 8.49 (2.35, 0.28, 2.03, 2.88, 0.95), leg III missing, leg IV: 7.38 (2.15, 0.26, 1.84, 2.43, 0.70); tibia I L/d: 45. Eye inter-distances and diameters: PME–PME 0.12, PME 0.08, PME–ALE 0.02, AME absent. Sternum width/length: 0.51/0.48. Habitus as in Fig. 13E, F. Carapace yellowish, with a pair of curved median bands; sternum yellowish. Legs yellowish, without darker rings. Opisthosoma yellowish, with dorsal and lateral black spots. Thoracic furrow absent. Clypeus unmodified. Chelicerae (Fig. 13D) with a pair of proximo-lateral apophyses and a pair of long, curved distal apophyses (distance between tips: 0.49). Palp as in Fig. 12A, B; trochanter with ventral apophysis (as long as wide; arrow in Fig. 12A) and small retrolateral apophysis (arrow 1 in Fig. 12B); femur with distinct ventral protrusion (arrow 2 in Fig. 12B); procursus simple proximally but complex distally, with narrow sclerite and teeth on prolatero-subdistal part (arrow 1 in Figs 12C, 18K), distal membranous process (arrow 2 in Figs 12C, 18K), and dorso-distal spine (arrow 3 in Figs 12C, 18K); bulb (Fig. 13C) with hooked apophysis and simple embolus. Retrolateral trichobothria on tibia I at 7% proximally; legs with short vertical setae on metatarsi; tarsus I with 16 distinct pseudosegments.

**Female** (***paratype***, IZCAS-Ar44976): Similar to male, habitus as in Fig. 13G, H. Total length 2.13 (2.21 with clypeus), carapace 0.68 long, 0.70 wide, opisthosoma 1.45 long, 1.00 wide; tibia I: 2.91; tibia I L/d: 36. Eye inter-distances and diameters: PME–PME 0.11, PME 0.07, PME–ALE 0.02, AME absent. Sternum width/length: 0.55/0.53. Epigyne (Figs 13A, 21C) simple and flat, anteriorly slightly sclerotized, with dark internal shade and a pair of postero-lateral pockets 0.46 apart. Vulva (Figs 13B, 21D) with ridge-shaped anterior arch, a pair of nearly triangular pore plates bearing distinct teeth, and four distinct median teeth (arrows in Figs 13B, 21D).

##### Variation.

Tibia I in the other three female paratypes (IZCAS-Ar44977–79): 2.25, 2.47, 2.55.

##### Habitat.

The species was found in the leaf litter.

##### Distribution.

China (Yunnan, type locality; Fig. 1).

#### ﻿ Belisana yongsheng

Taxon classificationAnimaliaAraneaePholcidae

Zhang, Li & Yao sp. nov.

90ACB0CE-FE40-536E-B1A6-F097C2532A52

https://zoobank.org/0996FDCD-9FEE-4635-B5D3-D9B14F913191

**Figure 14.**
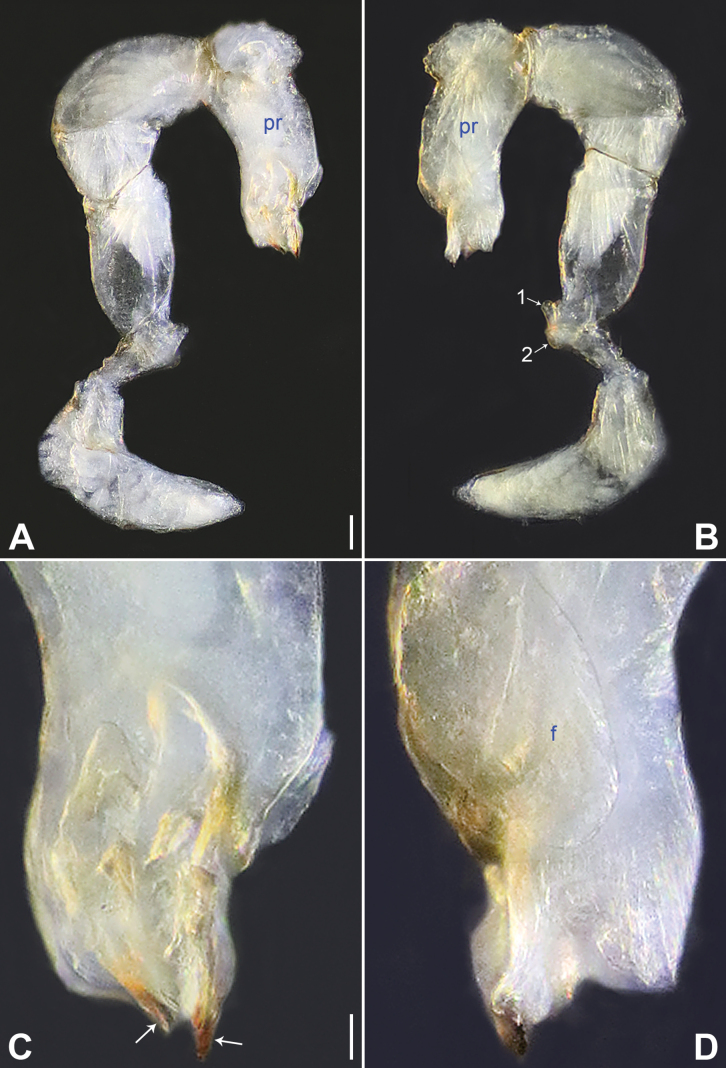
*Belisanayongsheng* sp. nov., holotype male **A, B** palp (**A** prolateral view **B** retrolateral view, arrow 1 points at ventral apophysis, arrow 2 points at retrolatero-ventral apophysis) **C, D** distal part of procursus (**C** prolateral view, arrows point at two sclerotized distal apophyses **D** retrolateral view). Abbreviations: f = flap, pr = procursus. Scale bars: 0.10 (**A, B**); 0.02 (**C, D**).

**Figure 15.**
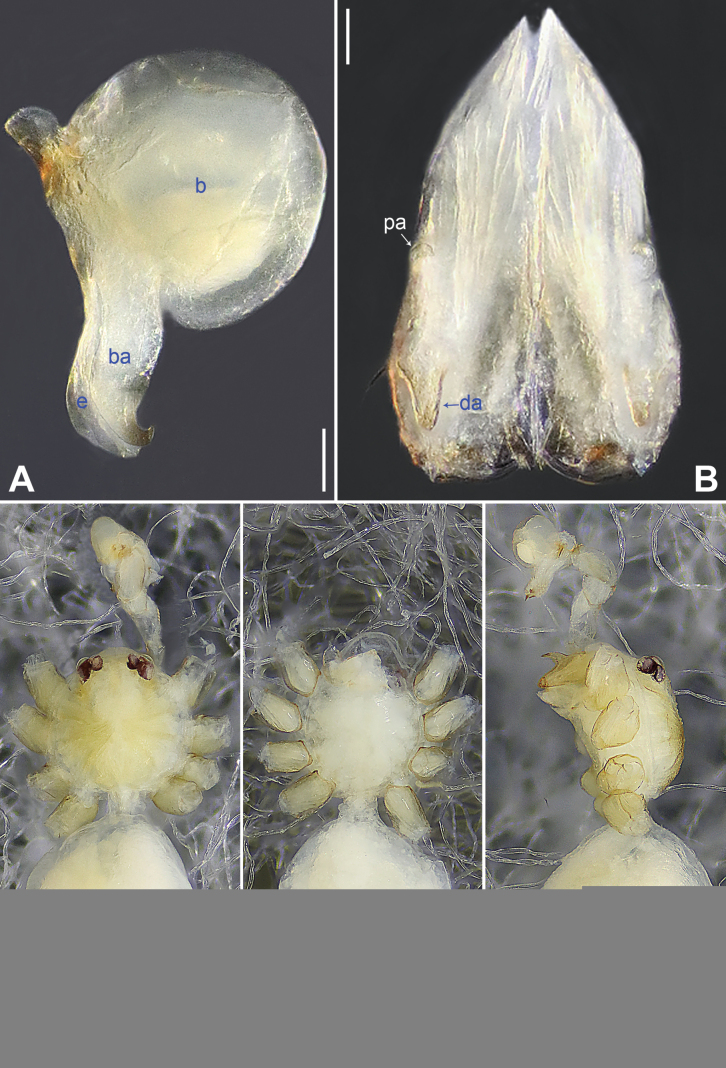
*Belisanayongsheng* sp. nov., holotype male **A** bulb, prolateral view **B** chelicerae, frontal view **C–E** habitus (**C** dorsal view **D** ventral view **E** lateral view). Abbreviations: b = bulb, ba = bulbal apophysis, da = distal apophysis, e = embolus, pa = proximo-lateral apophysis. Scale bars: 0.05 (**A, B**), 0.30 (**C–E**).

Figs 14 , 15 , 19A, B

##### Type material.

***Holotype*** ♂ (IZCAS-Ar44980), Yinhe Cave (26°27.127'N, 101°6.482'E, 2012 m), Renhe Town, Yongsheng County, Lijiang, **Yunnan**, **China**, 03/08/2010, C Wang, Q Zhao & L Lin leg.

##### Etymology.

The specific name refers to the type locality, which is a noun in apposition.

##### Diagnosis.

The new species resembles *B.halongensis* Yao, Pham & Li, 2015 (Yao et al. 2015: 8, figs 16A–D, 17A–D, 18A–D) by having similar male chelicerae and bulbal apophyses (Fig. 15A, B), but can be distinguished by differences in males: procursus with two sclerotized distal apophyses (arrows in Figs 14C, 19A vs one prolatero-ventral sclerite), retrolateral flap (Figs 14D, 19B vs absent) and without prolatero-distal membranous lamella (Figs 14C, 19A vs present).

##### Description.

**Male** (***holotype***): Total length 1.74 (1.79 with clypeus), carapace 0.58 long, 0.65 wide, opisthosoma 1.16 long, 0.80 wide. Leg I: 11.43 (3.10, 0.26, 3.03, 3.76, 1.28), leg II: 7.94 (2.25, 0.24, 1.98, 2.55, 0.92), leg III: 5.65 (1.60, 0.21, 1.38, 1.82, 0.64), leg IV: 7.29 (2.09, 0.23, 1.80, 2.48, 0.69); tibia I L/d: 47. Eye inter-distances and diameters: PME–PME 0.12, PME 0.07, PME–ALE 0.02, AME absent. Sternum width/length: 0.51/0.46. Habitus as in Fig. 15C–E. Carapace yellowish, with brownish radiating marks; sternum yellowish. Legs whitish, without darker rings. Opisthosoma yellowish, without spots. Thoracic furrow absent. Clypeus unmodified. Chelicerae (Fig. 15B) with a pair of proximo-lateral apophyses and a pair of distal apophyses (distance between tips: 0.19). Palp as in Fig. 14A, B; trochanter with ventral apophysis (as long as wide, arrow 1 in Fig. 14B) and retrolatero-ventral apophysis (arrow 2 in Fig. 14B); procursus simple proximally but complex distally, with two sclerotized distal apophyses (arrows in Figs 14C, 19A) and nearly D-shaped retrolateral flap (Figs 14D, 19B); bulb (Fig. 15A) with hooked apophysis and simple embolus. Retrolateral trichobothria on tibia I at 11% proximally; legs with short vertical setae on metatarsi; tarsus I with 20 distinct pseudosegments.

**Female**: Unknown.

##### Habitat.

The species was found in the dark zone inside the cave.

##### Distribution.

China (Yunnan, type locality; Fig. 1).

#### ﻿ Belisana yunnan

Taxon classificationAnimaliaAraneaePholcidae

Zhang, Li & Yao sp. nov.

1BB74B62-D952-5B7A-88A8-9E7FCBC129FE

https://zoobank.org/757F74E0-B68C-47E6-9260-5061A26D54A4

**Figure 16.**
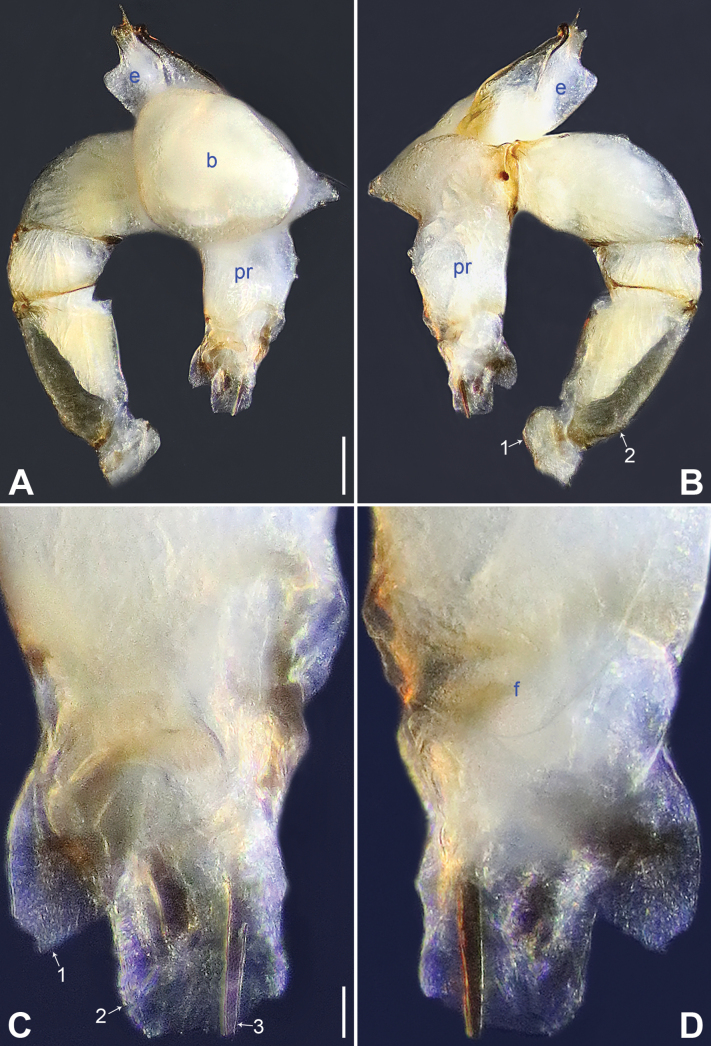
*Belisanayunnan* sp. nov., holotype male **A, B** palp (**A** prolateral view **B** retrolateral view, arrow 1 points at ventral apophysis, arrow 2 points at retrolatero-proximal protrusion) **C, D** distal part of procursus (**C** prolateral view, arrow 1 points at prolatero-ventral membranous lamella, arrow 2 points at distal membranous lamella, arrow 3 points at distal spine **D** retrolateral view). Abbreviations: b = bulb, e = embolus, f = flap, pr = procursus. Scale bars: 0.10 (**A, B**); 0.02 (**C, D**).

**Figure 17.**
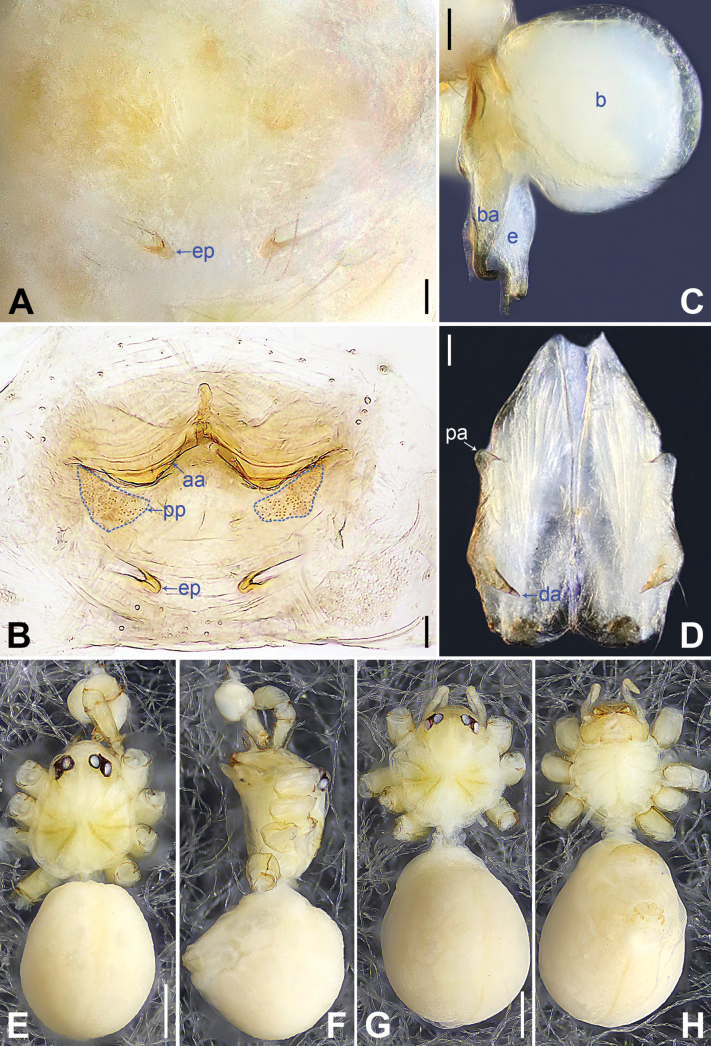
*Belisanayunnan* sp. nov., holotype male (**C–F**) and paratype female (**A, B, G, H**) **A** epigyne, ventral view **B** vulva, dorsal view **C** bulb, prolateral view **D** chelicerae, frontal view **E–H** habitus (**E, G** dorsal view **F** lateral view **H** ventral view). Abbreviations: aa = anterior arch, b = bulb, ba = bulbal apophysis, da = distal apophysis, e = embolus, ep = epigynal pocket, pa = proximo-lateral apophysis, pp = pore plate. Scale bars: 0.05 (**A–D**); 0.30 (**E–H**).

Figs 16 , 17 , 19C, D , 21E, F

##### Type material.

***Holotype*** ♂ (IZCAS-Ar44981) and ***paratype*s** 2♂ (IZCAS-Ar44982–83) 3♀ (IZCAS-Ar44984–86), Xianren Cave (24°13.929'N, 98°25.563'E, 1636 m), Luxi, Dehong, **Yunnan**, **China**, 24/08/2010, C Wang, Q Zhao & L Lin leg.

##### Etymology.

The specific name refers to the type locality, which is a noun in apposition.

##### Diagnosis.

The new species resembles *B.yuhaoi* Yang & Yao, 2023 (Yang et al. 2023: 178, figs 2A, B, 3A–D, 4A–H) by having similar bulbal apophyses and epigyne (Figs 17A, C, 21E), but can be distinguished by differences in males: tips of cheliceral distal apophyses closer to each other (Fig. 17D vs widely separated), procursus without sclerotized dorso-subdistal apophysis (Figs 16C, 19C vs present) and with nearly half-round retrolateral flap (Figs 16D, 19D vs angular); differences in females: epigynal pockets closer to each other (Figs 17A, B, 21E, F vs widely separated), pore plates nearly isosceles triangle-shaped (Figs 17B, 21F vs scalene triangle-shaped).

##### Description.

**Male** (***holotype***): Total length 1.90 (1.98 with clypeus), carapace 0.79 long, 0.71 wide, opisthosoma 1.11 long, 0.87 wide. Leg I: 18.71 (4.85, 0.56, 4.75, 7.05, 1.50), leg III: 9.35 (2.66, 0.30, 2.25, 3.25, 0.89), legs II and IV missing; tibia I L/d: 59. Eye inter-distances and diameters: PME–PME 0.11, PME 0.08, PME–ALE 0.03, AME absent. Sternum width/length: 0.63/0.60. Habitus as in Fig. 17E, F. Carapace yellowish, with brown radiating marks; sternum yellowish. Legs whitish, without darker rings. Opisthosoma yellowish, without spots. Thoracic furrow absent. Clypeus unmodified. Chelicerae (Fig. 17D) with a pair of proximo-lateral apophyses and a pair of curved distal apophyses (distance between tips: 0.19). Palp as in Fig. 16A, B; trochanter with ventral apophysis (as long as wide, arrow 1 in Fig. 16B); femur with tiny retrolatero-proximal protrusion (arrow 2 in Fig. 16B); procursus simple proximally but complex distally, with prolatero-ventral membranous lamella (arrow 1 in Figs 16C, 19C) bearing median sclerotized part, distal membranous lamella (arrow 2 in Figs 16C, 19C) bearing median sclerotized part, distal spine (arrow 3 in Figs 16C, 19C), and nearly half-round retrolateral flap (Figs 16D, 19D); bulb (Fig. 17C) with hooked apophysis and simple embolus. Retrolateral trichobothria on tibia I at 14% proximally; legs with short vertical setae on metatarsi; tarsus I with 21 distinct pseudosegments.

**Figure 18.**
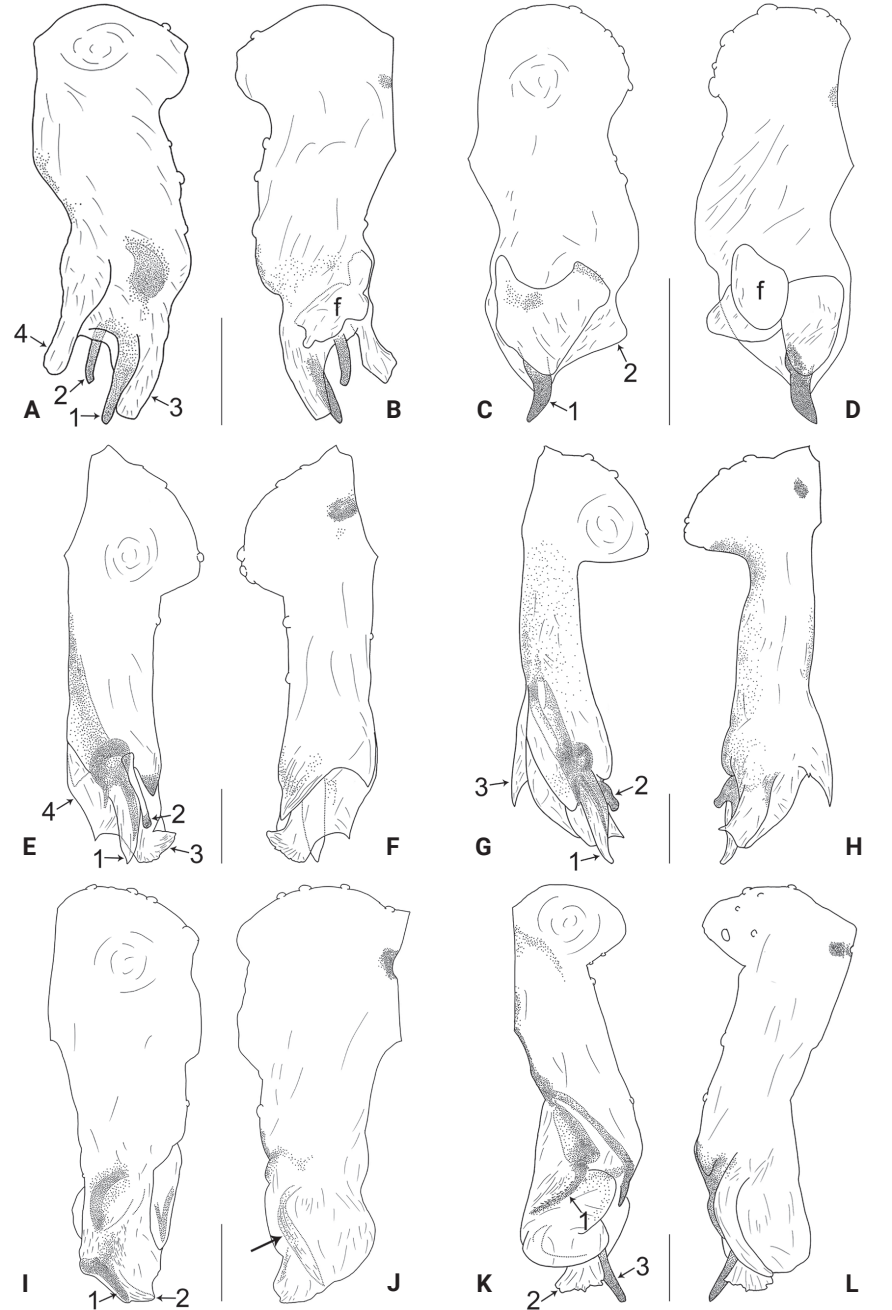
Procursus in prolateral and retrolateral views (arrows point at same structures as photos of each species) **A, B***Belisanahonghe* sp. nov. **C, D***B.jiuxiang* sp. nov. **E, F***B.lincang* sp. nov. **G, H***B.luxi* sp. nov. **I, J***B.tengchong* sp. nov. **K, L***B.tongi* sp. nov. Abbreviation: f = flap. Scale bars: 0.10.

**Figure 19.**
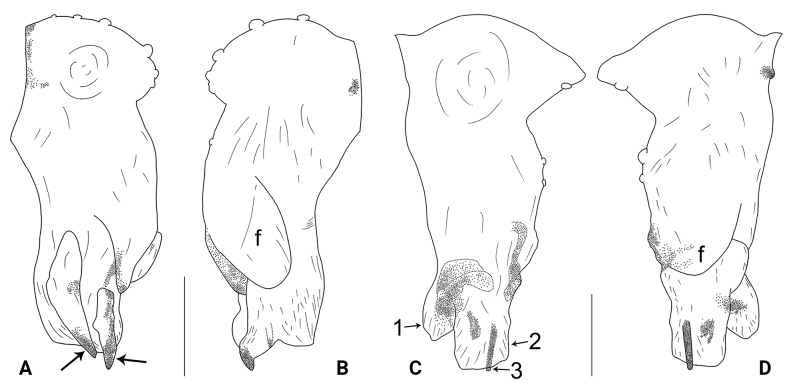
Procursus in prolateral and retrolateral views (arrows point at same structures as photos of each species) **A, B***Belisanayongsheng* sp. nov. **C, D***B.yunnan* sp. nov. Abbreviation: f = flap. Scale bars: 0.10.

**Figure 20.**
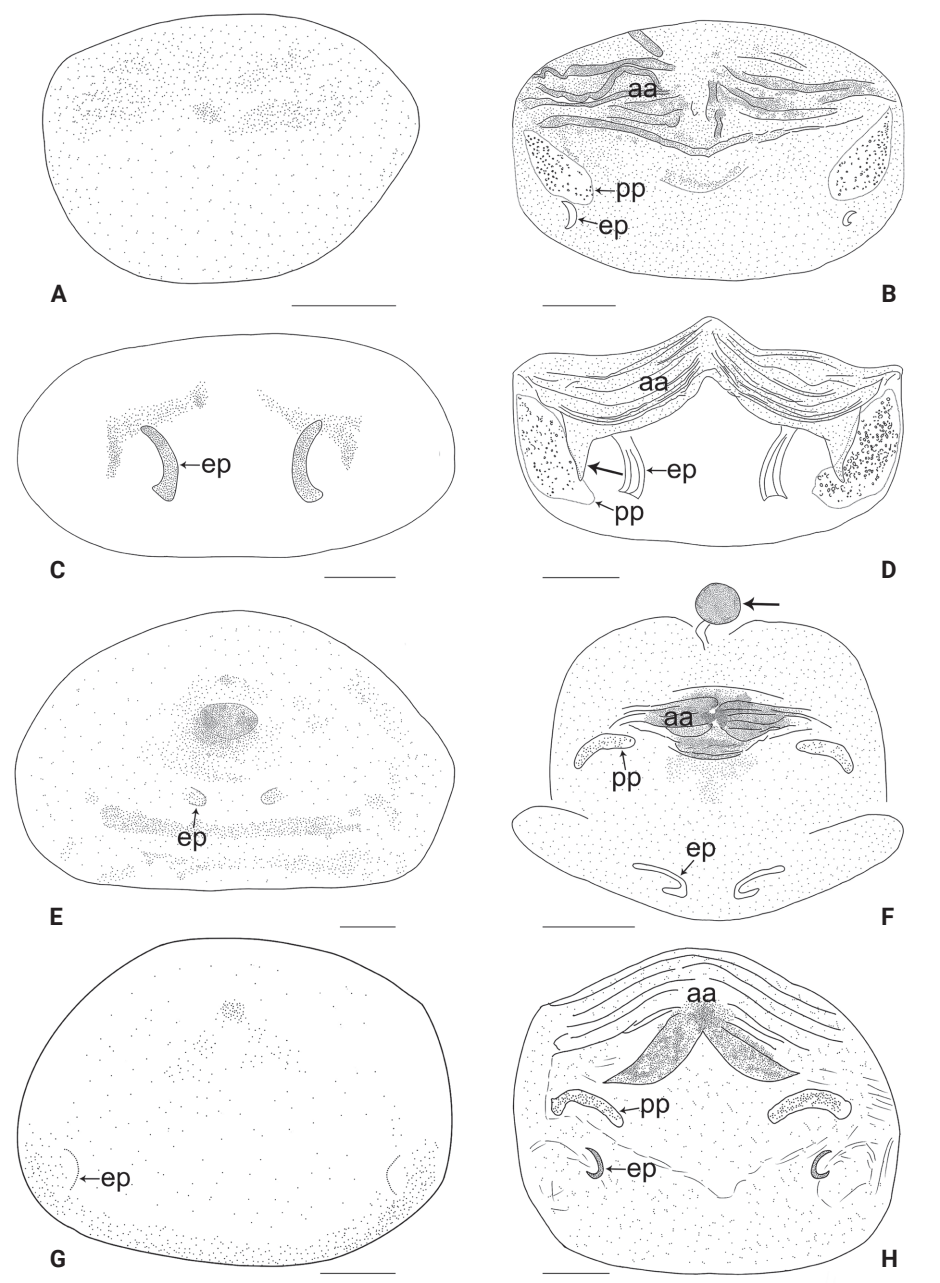
Female genitalia in ventral and dorsal views **A, B***Belisanahonghe* sp. nov. **C, D***B.jiuxiang* sp. nov., arrow points at lateral sclerite **E, F***B.lincang* sp. nov., arrow points at sac-like structure **G, H***B.luxi* sp. nov. Abbreviations: aa = anterior arch, ep = epigynal pocket, pp = pore plate. Scale bars: 0.10.

**Figure 21.**
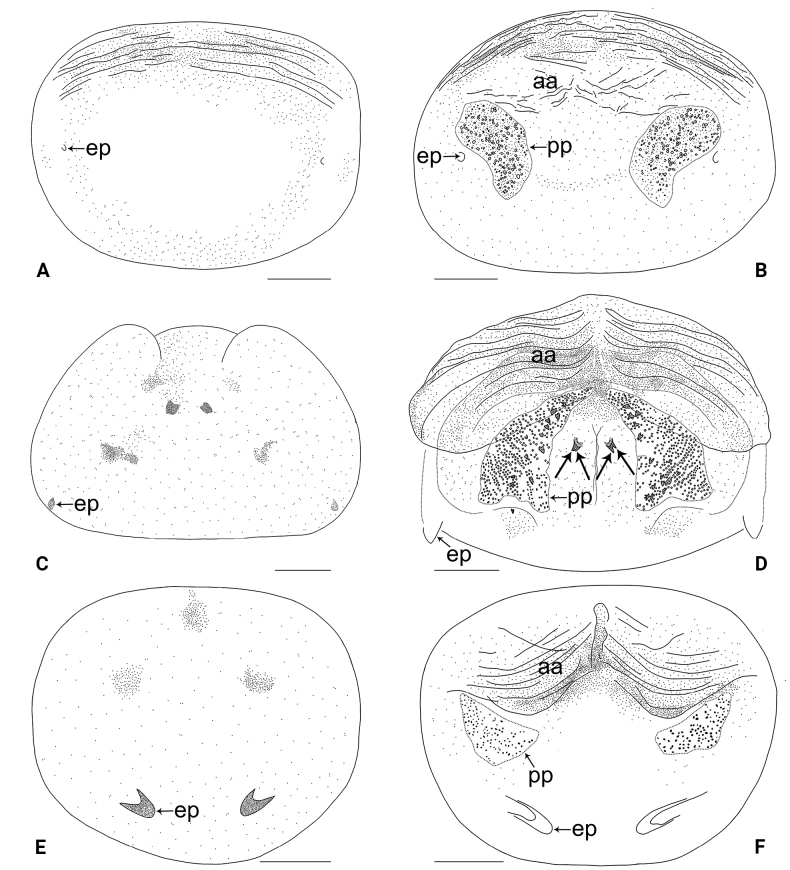
Female genitalia in ventral and dorsal views **A, B***Belisanatengchong* sp. nov. **C, D***B.tongi* sp. nov., arrows point at four distinct median teeth **E, F***B.yunnan* sp. nov. Abbreviations: aa = anterior arch, ep = epigynal pocket, pp = pore plate. Scale bars: 0.10.

**Female** (***paratype***, IZCAS-Ar44984): Similar to male, habitus as in Fig. 17G, H. Total length 2.14 (2.20 with clypeus), carapace 0.78 long, 0.77 wide, opisthosoma 1.36 long, 1.00 wide; tibia I: 3.88; tibia I L/d: 52. Eye inter-distances and diameters: PME–PME 0.13, PME 0.07, PME–ALE 0.02. Sternum width/length: 0.62/0.56. Epigyne (Figs 17A, 21E) simple and flat, anteriorly slightly sclerotized, with a pair of postero-median pockets 0.12 apart. Vulva (Figs 17B, 21F) with ridge-shaped anterior arch and a pair of nearly triangular pore plates.

##### Variation.

Tibia I in two male paratypes (IZCAS-Ar44982–83): 5.38, 6.73. Tibia I in the other two female paratypes (IZCAS-Ar44985–86): 3.72, 3.85.

##### Habitat.

The species was found in the dark zone inside the cave.

##### Distribution.

China (Yunnan, type locality; Fig. 1).

## ﻿Discussion

Altogether, including the eight species described in this paper, there are now 70 species of *Belisana* in China, representing 45% of the global total of the genus. Within China, 31 species (44%) were found in Yunnan. Table 1 shows that within Yunnan, the species count from Xishuangbanna (19 species) far outstrips those of the Hengduan Mountains (six species) and Yunnan-Guizhou Plateau (six species), both of which are geographically larger in area than the Xishuangbanna (Fig. 1). It is easy to speculate on the reasons for the apparent disparity among the three datasets: field collections in Xishuangbanna have been more frequent than in the Hengduan Mountains and the Yunnan-Guizhou Plateau. Although we do not have hard figures in terms of man-hours, it is our general impression that more intensive efforts have been expended in collecting spiders in Xishuangbanna than in the two other localities, partly because fogging was carried out in Xishuangbanna and not elsewhere. In fact, among the 19 species from Xishuangbanna, seven species were collected by fogging. In conclusion, it should not be unreasonable to hypothesize that more new species of *Belisana* may be discovered in the Hengduan Mountains and Yunnan-Guizhou Plateau when a more concerted effort is made to collect from both areas, which differ from Xishuangbanna by their subtropical climate, larger geographic expanse, and by a higher prevalence of karst landforms.

## Supplementary Material

XML Treatment for Belisana

XML Treatment for Belisana honghe

XML Treatment for Belisana jiuxiang

XML Treatment for Belisana lincang

XML Treatment for Belisana luxi

XML Treatment for Belisana tengchong

XML Treatment for Belisana tongi

XML Treatment for Belisana yongsheng

XML Treatment for Belisana yunnan

## ﻿Additional information

### Author ORCIDs

Ludan Zhang https://orcid.org/0000-0001-5657-751X

Shuqiang Li https://orcid.org/0000-0002-3290-5416

Zhiyuan Yao https://orcid.org/0000-0002-1631-0949

### Data availability

All of the data that support the findings of this study are available in the main text.
